# Elevating serotonin pre-partum alters the Holstein dairy cow hepatic adaptation to lactation

**DOI:** 10.1371/journal.pone.0184939

**Published:** 2017-09-18

**Authors:** Samantha R. Weaver, Allan S. Prichard, Noah L. Maerz, Austin P. Prichard, Elizabeth L. Endres, Lorenzo E. Hernández-Castellano, Matthew S. Akins, Rupert M. Bruckmaier, Laura L. Hernandez

**Affiliations:** 1 Department of Dairy Science, University of Wisconsin-Madison, Madison, Wisconsin, United States of America; 2 Veterinary Physiology, Vetsuisse Faculty, University of Bern, Bern, Switzerland; 3 Department of Dairy Science, University of Wisconsin-Marshfield, Marshfield, Wisconsin, United States of America; University of Illinois, UNITED STATES

## Abstract

Serotonin is known to regulate energy and calcium homeostasis in several mammalian species. The objective of this study was to determine if pre-partum infusions of 5-hydroxytryptophan (5-HTP), the immediate precursor to serotonin synthesis, could modulate energy homeostasis at the level of the hepatocyte in post-partum Holstein and Jersey dairy cows. Twelve multiparous Holstein cows and twelve multiparous Jersey cows were intravenously infused daily for approximately 7 d pre-partum with either saline or 1 mg/kg bodyweight of 5-HTP. Blood was collected for 14 d post-partum and on d30 post-partum. Liver biopsies were taken on d1 and d7 post-partum. There were no changes in the circulating concentrations of glucose, insulin, glucagon, non-esterified fatty acids, or urea nitrogen in response to treatment, although there were decreased beta-hydroxybutyrate concentrations with 5-HTP treatment around d6 to d10 post-partum, particularly in Jersey cows. Cows infused with 5-HTP had increased hepatic serotonin content and increased mRNA expression of the serotonin 2B receptor on d1 and d7 post-partum. Minimal changes were seen in the hepatic mRNA expression of various gluconeogenic enzymes. There were no changes in the mRNA expression profile of cell-cycle progression marker cyclin-dependent kinase 4 or apoptotic marker caspase 3, although proliferating cell nuclear antigen expression tended to be increased in Holstein cows infused with 5-HTP on d1 post-partum. Immunofluorescence assays showed an increased number of CASP3- and Ki67-positive cells in Holstein cows infused with 5-HTP on d1 post-partum. Given the elevated hepatic serotonin content and increased mRNA abundance of *5HTR2B*, 5-HTP infusions may be stimulating an autocrine-paracrine adaptation to lactation in the Holstein cow liver.

## Introduction

During early lactation, the dairy cow must make dramatic and rapid metabolic adaptions to prepare for the onset of copious milk production. At this time, the dairy cow is vulnerable to development of several transition period disorders. Hypocalcemia, as well as ketosis, are key players in the transition period negative energy balance. Negative energy balance results from high energy demands for maintenance of milk production coupled with inadequate dietary energy intake. One study found that each 0.1 mM increase in beta-hydroxybutyrate (BHBA) at first subclinical ketosis-positive test resulted in an 0.5 kg/day decrease in milk production for the first 30 days of lactation, as well as increased displaced abomasum and removal from the herd [1]. Together, ketosis and hypocalcemia represent two potentially devastating insults to the lactating dairy cow. As such, a common therapy could represent a powerful solution within the dairy industry. Serotonin has the potential to serve as an essential tool in the prevention of transition-related dairy cow diseases.

Serotonin is a potent regulator of both calcium homeostasis and energy homeostasis during lactation in both rodent and dairy cow models [2, 3]. Serotonin is a modified amino acid: L-tryptophan is converted to 5-hydroxytryptophan (5-HTP) using the rate-limiting enzyme tryptophan hydroxylase (TPH). There are two isoforms of TPH: one in the periphery and the other in the central nervous system. Since serotonin cannot cross the blood-brain barrier, mammalian serotonergic pools are effectively isolated [4]. In the central nervous system, the isoform TPH2 hydroxylates L-tryptophan to 5-HTP, which is then decarboxylated by amino acid decarboxylase to yield 5-hydroxytryptamine, or serotonin. This serotonin can then bind its 14 receptor isoform subtypes in the nervous system and periphery. Central serotonin regulates a variety of processes, including mood [5] and suppression of food intake [2, 6]. Serotonin produced in the periphery, using the isoform TPH1, affects energy homeostasis [7]. Much of the work on serotonin and energy balance has been performed in rodents, highlighting specific organs and key serotonin receptors. For example, mice with TPH1 deficiency in adipose tissue were resistant to high fat diet-induced weight gain and glycemic control was improved compared to controls [8]. In mouse adipocytes, gut-derived serotonin signaling through the serotonin receptor 2B (*HTR2B*) favored lipolysis, while *HTR2B* signaling in hepatocytes promoted gluconeogenesis [9]. During pregnancy in mice, serotonin expands beta cell mass and proliferation through the *HTR2B* receptor [10]. In an autocrine-paracrine fashion, pancreatic islet serotonin attunes glucose responsiveness of beta cells to increase glucose-stimulated insulin secretion through the *HTR2B* at mid-gestation, and reduces beta cell mass through *HTR1D* at the end of gestation in mice [11]. Blood serotonin is substantially elevated in diet-induced obese mice [12]. In the liver, serotonin is also implicated in hepatic regeneration: low serotonin levels preceding liver resection in human patients predicted postoperative liver dysfunction [13] and signaling through *HTR2A* and *2B* rescued the age-associated decline in hepatic regeneration following liver resection [14]. Finally, mRNA expression of various gluconeogenic and glycolytic enzymes in the liver and glucose transporters in the mammary gland were elevated in rats fed a diet supplemented with 5-HTP when compared to controls [15].

While there is a growing body of literature surrounding serotonin and energy homeostasis [7, 16], very little work has been performed in ruminant models. Ruminants have enormous demands for hepatic gluconeogenesis [17] in order to provide sufficient precursors for lactose synthesis in milk [18]. Preliminary studies have established that serotonin receptor expression is dynamic in the liver of transition period dairy cows [19]. Additionally, work by Watanabe and coauthors (2014) have shown that intravenous injections of serotonin after fasting for 24 hours to wethers elevated circulating concentrations of glucose, insulin, triglyceride, and NEFA, likely through the *HTR1D*, establishing a precedent for serotonin’s role in energy status in ruminants [20]. The objective of this study was to establish whether increasing peripheral serotonin concentrations through infusion of the serotonergic precursor 5-HTP pre-partum would induce alterations in local, hepatic energy homeostasis in post-partum, multiparous dairy cows.

## Materials and methods

### Animals and experimental design

All of the experiments were performed under protocols approved by the Animal Care and Use Committee at the University of Wisconsin-Madison (Protocol number A01521). Information about animal housing and animal diets both pre- and post-partum are provided in [21]. Briefly, three weeks prior to their date of expected parturition, 12 multiparous Holstein (average lactation number 3.67 ± 0.43) and 12 multiparous Jersey (average lactation number 2.83 ± 0.37) dairy cows were transported to the Dairy Cattle Center on the University of Wisconsin-Madison campus from the Emmons Blaine Dairy Cattle Research Center (Arlington Dairy) approximately 23 miles outside of Madison, WI. Cows were transported via road trailer three weeks prior to their date of expected parturition and not handled for one full week in order to give the cows time to adapt to their new facilities and feeding schedule at the Dairy Cattle Center. Approximately seven days before the predicted date of parturition, cows were weighed and infused daily over the course of approximately 45 minutes with either 1 liter of saline or 1 liter of saline containing 1 mg/kg bodyweight of 5-HTP (Sigma Aldrich, St. Louis, MO, USA). We previously determined that this dose of 5-HTP was safe to administer to both late lactation [22] and transition period [21] dairy cows. In addition, this dose was sufficient to modify various metabolic parameters in late-lactation dairy cows [22]. The final treatment groups were Holstein cows infused with saline (H CON; *n* = 6) or saline + 5-HTP (H 5-HTP; *n* = 6) and Jersey cows infused with saline (J CON; *n* = 6) or saline + 5-HTP (J 5-HTP; *n* = 6). Holstein cows were infused for 5.67 ± 0.78 days (d) and Jersey cows were infused for 8.67 ± 1.53 d pre-partum leading up to the day of parturition (*P*>0.05) [21]. All cows were infused for at least 3 d with the exception of one H CON that was infused for 1 d and one J 5-HTP that was infused for 2 d. Previous work from our laboratory has shown that 2 d of serotonin infusion at 1.0 mg/kg is sufficient to elevate circulating serotonin concentrations [22]. The day following parturition (d1) and on d7 post-partum, cows were subjected to a liver biopsy. Blood was collected daily from the day of parturition (d0) through d15 and on d30 post-partum. This study was originally designed and powered to examine calcium homeostasis in response to 5-HTP infusion during the transition period. Results from that portion of the study are published in [21]. The data presented herein represent a secondary objective to examine hepatic response to 5-HTP infusion and as such, blood and tissue sampling times are secondary to the main endpoint of calcium homeostasis.

### Sample collection

A comprehensive explanation of blood collection methodology is detailed in work by Weaver and colleagues [21]. Briefly, approximately 7 d pre-partum, a jugular catheter (Abbocath-T Subclavian I.V. 14g×5 1/2” Catheter, Hospira, Lake Forest, IL, USA) was inserted into each cow and subsequently flushed with heparinized saline every 8 hours for the entirety of the study. The jugular catheter was used for infusion of the saline or saline + 5-HTP until the day of parturition. Following parturition, blood was collected daily from the coccygeal vein. Whole blood was collected each morning following parturition at approximately 8 a.m. Feed was distributed in the dairy at approximately the same time (between 8 and 9 a.m.), although blood samples were not taken relative to feeding per se, but rather at a consistent time each morning. To isolate the serum fraction, blood was collected into 10mL BD Vaccutainer Serum Plus Tubes (367820, BD, Franklin Lakes, NJ, USA). For the plasma fraction, Lithium Heparin 158 USP Units Plus Blood Collection Tubes (367880, BD, Franklin Lakes, NJ, USA) were used. The serum or plasma fractions were collected following centrifugation at 3000 x rpm for 20 min at 4°C and stored at -80°C until analysis.

Percutaneous liver biopsy samples were collected on d1 and d7 post-partum between 10 am and 12 pm, depending on the number of cows that required biopsy on a given day. Prior to the surgery, cows were administered an intravenous sedative and analgesic cocktail (0.02 mg/kg butorphanol, Fort Dodge Laboratories (Fort Dodge, IA, USA), and 0.02 mg/kg xylazine, AnaSed (Shenandoah, IA, USA)). The surgical site was then prepared by alternating betadine surgical scrub (Veterinary, Purdue Products, Stamford, CT, USA) and 70% ethanol scrubs. Local anesthetic (10 mL of 5% lidocaine, Sparhawk Laboratories, Mission, KS, USA) was administered at the biopsy site. On d1 post-partum, the biopsy was taken from the third intercostal space counting forward from the tail-end of the cow. On d7 post-partum, the sample was acquired from the second intercostal space from the back. One piece of liver tissue weighing approximately 1.0 to 2.0 grams was snap-frozen in liquid nitrogen and stored at −80°C until RT-PCR and protein isolation. Another piece of tissue was placed in a cassette and fixed overnight in 4% paraformaldehyde, then transferred to 70% ethanol until paraffin embedding and sectioning. Immediately following each procedure, cows were monitored in a tie-stall for safe recovery from the sedative and analgesic cocktail, including respiration and heart rate. Tissue sites were monitored for signs of infection including swelling, redness, and presence of pus and general cow health (rectal temperature, affect) was monitored for 14 d following each surgery in close collaboration with University of Wisconsin-Madison veterinarians.

### Serum and plasma laboratory analyses

For all assays performed, a quality control (QC) was analyzed on each plate to assure the integrity of the assay. Glucose was analyzed in plasma using a glucose oxidase-peroxidase assay specific for glucose [22, 23]. The intra- and inter-assay CVs were 2.3% and 6.5%, respectively. Serum NEFA concentrations were determined using an enzymatic colorimetric assay (NEFA-HR (2), Wako Chemicals, Richmond, VA, USA), with intra- and inter-assay CVs of 2.3% and 7.7%. Plasma urea nitrogen (PUN) concentrations were measured using a colorimetric detection kit (K024-H1, Arbor Assays, Ann Arbor, MI, USA). The intra-assay CV was 1.2% and the inter-assay CV was 9.5%. Plasma concentrations of BHBA were measured enzymatically (RB1007, Randox Laboratories Ltd, Switzerland) with an intra-assay CV of 4.4% and inter-assay CV of 5.3%. Insulin and glucagon were both measured through d7 post-partum in plasma. Insulin was evaluated via radioimmunoassay with an intra-assay CV of 9.4% and inter-assay CV of 6.7%, as previously described [24]. Plasma glucagon was measured using a radioimmunoassay kit (GL-32K, Millipore, Zug, Switzerland). The intra-assay CV was 6.9% and the inter-assay CV was 4.7%.

### Liver protein isolation, protein assays, and histology

Protein was isolated from liver tissue using radioimmunoprecipitation buffer (RIPA) plus 10 μL/mL of Halt Protease and Phosphatase Inhibitors Cocktail (Thermo Scientific #78441). Protein concentrations were determined using the bicinchoninic acid assay (Pierce Chemicals #23227). Liver content of serotonin was determined following the manufacturer’s instructions using a Serotonin EIA Kit (IM1749, Immunotech, Beckman Coulter, Marseille Cedex 9, France), loading 50 μg of protein per sample. The intra-assay CV was 4.8%. As all samples were run on one plate, there was no inter-assay CV.

Tissue used for histology was fixed in 4% paraformaldehyde overnight at 4°C then transferred to 70% ethanol until dehydration with xylene and paraffin embedding. Paraffin blocks were sectioned at 5 μm. Cell proliferation in liver tissue was determined by immunolabeling with Ki67 antibody (Abcam, #ab5580, 1:1000) and cell apoptosis was evaluated using a Caspase 3 antibody (Cell Signaling Technology, #9661L, 1:300) overnight at 4°C following a 1 hour block in 5% horse serum blocking buffer. For Ki67-stained sections, a 0.1% preparation of Sudan Black (Sigma Aldrich #199664) in 70% ethanol was applied for 25 minutes at room temperature immediately following the block to prevent auto-fluorescence. Secondary antibody was incubated for 1 h at room temperature (1:250, Alexa Fluor 488 Goat Anti-Rabbit IgG (Life Technologies, #A11012)). Nuclei were visualized with 49, 6-diamidino-2-phenylindole (DAPI, 1:1000 in blue). Proliferating or apoptotic cell numbers were counted using ImageJ software (NIH version 1.8).

### Liver RNA extraction and quantitative real-time PCR

Total RNA was extracted from liver tissue using TRI-Reagent (Molecular Research) and the Tissue Master 125 Homogenizer (Omni International). RNA was quantified using a Nanodrop Spectrophotometer 200C (Thermo Scientific) with an average yield of 705 ± 46 ng/μL. The purity of the RNA (as evaluated by the A260/A280 ratio) was 1.89 ± 0.02. RNA was reverse transcribed (1 μg) to cDNA using Bio-Rad iScript Reverse Transcription Supermix (#1708840) following the manufacturer’s instructions. Quantitative RT-PCR was conducted with the CFX96 Touch Real-Time PCR Detection System (Bio-Rad). Reaction mixtures and cycling conditions were performed as previously described [15]. All primers were designed to span exon-exon junctions and for an optimal annealing temperature of 60°C. Amplification efficiencies of primers were accepted within a range of 95 to 105% efficiency and primer specificity was assessed by the presence of a single temperature dissociation peak, eliminating and redesigning any primers with indication of secondary structures. Hepatic mRNA was evaluated for tryptophan hydroxylase 1 (*TPH1*) and serotonin reuptake transporter (*SERT*) to measure serotonin synthesis and reuptake, serotonin receptors 1D (*5HTR1D*), 2A (*5HTR2A*), 2B (*5HTR2B*), and 7 (*5HTR7*) to evaluate local hepatic serotonergic signaling, cyclin D1 (*CCND1*), cyclin-dependent kinase 4 (*CDK4*), and proliferating cell nuclear antigen (*PCNA*) for cell cycle progression, and caspase 3 (*CASP3*) for apoptosis. Additionally, mitochondrial phosphoenolpyruvate carboxykinase (*PEPCK2*), pyruvate carboxylase (*PC*), and glucose 6-phosphatase (*G6P*) mRNA abundance was evaluated for gluconeogenesis. Primer sequences can be found in Table 1. The geometric mean of ribosomal protein 15 (*RPS15*) and cyclophilin A (*CYCLOA*) was used as the housekeeping parameter, and analysis was conducted using the 2^−ΔΔCt^ method [25]. The housekeeping genes *RPS15* and *CYCLOA* were chosen due to the narrow range of CT values encompassing all samples (21.81 ± 0.18, 24.08 ± 0.30 and 22.91 ± 0.22 for RPS15, CYCLOA, and the geometric mean of the two, respectively). In order to examine the difference in gene expression between breeds, all treatment groups were analyzed using H CON d1 liver CT values as the internal controls. When examining the effect of treatment within breed, H CON d1 CT values and J CON d1 CT values were used for Holsteins and Jerseys, respectively.

**Table 1:** Primer sequences for genes quantified by real-time PCR.

Gene	GeneBank Accession ID	Range of CT values	Sequence
*RPS15*	NM_001024541	20.41 to 23.42	Forward 5’-CGCGACATGATCATTCTACC-3’ Reverse 3’-TTACTTGAGGGGGATGAAG-5’
*CYCLOA*	NM_178320	21.97 to 25.84	Forward 5’-CACCGTGTTCTTCGACATCG-3’ Reverse 3’-ACAGCTCAAAAGAGACGCGG-5’
*TPH1*	XM_010822787	26.39 to 30.24	Forward 5’- AGAGAATTTACCAAGACAATCAAG-3’ Reverse 3’- CTTAGCAAGGGCATCACTGAC-5’
*SERT*	NM_174609	34.02 to 40.06	Forward 5’- GAAGCTGTTGGAGGAGTTCG-3’ Reverse 3’- CCAGCAGATCTTRCCAGAACC-5’
*5HTR1D*	XM_015462457	24.06 to 36.69	Forward 5’-CCTCCAACAGATCCCTGAATG-3’ Reverse 3’-CAGAGCAATGACACAGAGATGCA-5’
*5HTR2A*	NM_001001157	25.01 to 38.31	Forward 5’- TCCTGTTTGTGGTGATGTGG-3’ Reverse 3’- GGTTGACTGCTGAGGAGAGG-5’
*5HTR2B*	XM_010802399	24.49 to 32.73	Forward 5’-AAACAAGCCACCTCAACGCCT-3’ Reverse 3’-TCCCGAAATGTCTTATTGAAGAG-5’
*5HTR7*	XM_ 580794	24.64 to 33.68	Forward 5’-GTTTTATATCCCCATGTCCGTCA-3’ Reverse 3’-TTTGCACACTCCTCTACCTCCT-5’
*CCND1*	NM_001046273	27.93 to 33.54	Forward 5’- CGTGACGTGAACATCTGAGG-3’ Reverse 3’- AGACGGAAGGGAAAAAGAGC-5’
*CDK4*	NM_001037594	22.94 to 30.52	Forward 5’-AAGTGGTGGGACAGTCAAGC-3’ Reverse 3’-CTGCAAAGATACAGCCAACG-5’
*PCNA*	NM_001034494	24.34 to 31.96	Forward 5’-CCCTTGAAGGATGAAAATGG-3’ Reverse 3’- TCCCTAACACACCAGGAAGG-5’
*CASP3*	NM_001077840	25.18 to 31.45	Forward 5’-TACTTTTCCTGGCGAAATGC-3’ Reverse 3’-TTGCATGAAAAGCAGAATCG-5’
*PEPCK2*	NM_001205594	23.74 to 33.06	Forward 5’-TGGCCATGATGAACCCTACTC-3’ Reverse 3’-GTCAAATTTCATCCAGGCATA-5’
*PC*	NM_177946	25.47 to 39.79	Forward 5’-CCACGAGTTCTCCAACACCT-3’ Reverse 3’-TTCTCCTCCAGCTCCTCGTA-5’
*G6P*	NM_001076124	29.79 to 37.65	Forward 5’-TGATGGACCAAGAAAGATCCAGGG-3’ Reverse 3’-TAGGGATTGACCTCACTGGCCCTCTT-5’

All primers were designed using the Primer3 Input v.0.4.0. (http://bioinfo.ut.ee/primer3-0.4.0/). GeneBank accession numbers are listed beside the primer name. Primer sequences are presented as 5’ to 3’ (forward) and 3’ to 5’ (reverse). *RPS15* –ribosomal protein 15; *CYCLOA*–cyclophilin A; *TPH1* –tryptophan hydroxylase 1; *SERT*–serotonin reuptake transporter; *5HTR1D* –serotonin receptor 1d; *5HTR2A* –serotonin receptor 2a; *5HTR2B* –serotonin receptor 2b; *5HTR7* –serotonin receptor 7; *CCND1* –cyclin D1; *CDK4* –cyclin-dependent kinase 4; *PCNA*–proliferating cell nuclear antigen; *CASP3* –caspase 3; *PEPCK2* –phosphoenolpyruvate carboxykinase 2 (mitochondrial); *PC*–pyruvate carboxylase; *G6P* –glucose 6 phosphatase

### Statistical analysis

Analysis of all data was completed using a linear mixed model with repeated measures where necessary with SAS software (version 9.3; SAS Institute Inc., Cary, NC). Pre-partum and post-partum time periods as well as within-treatment or within-breed effects were analyzed separately. The model used was Y = T + B + T*B + D + D*T + D*B + D*T*B + e, where T was the fixed effect of treatment with saline or 5-HTP, B was the fixed effect of the breed (Holstein or Jersey), D was the fixed effect of the day relative to parturition (DRP), with parturition as day (0). The random effect of Cow within treatment was considered the error term for all effects that did not include the day (D) effect. In order to account for auto-correlated errors due to repeated measurements on the same experimental unit, Cow(T), an ar(1) error structure was used within the SAS MIXED procedure. When sampling time points were unevenly spaced, the covariance structure sp(pow) was used instead of ar(1). The residuals from the model were analyzed with a normality test and corrected using ranks, when appropriate. Each parameter was evaluated for extreme influential data points and outliers, which were removed when identified. Nonsignificant model terms were removed in a backwards stepwise elimination using a cut-off of P<0.05 for elimination. In addition to running the overall model for each parameter, we analyzed the data within only Holstein cows or only Jersey cows in order to examine the effect of treatment within a breed. In addition, we analyzed datasets consisting of only cows treated with saline or only cows treated with 5-HTP in order to evaluate the effect of breed within each treatment. For all analyses, differences between means were considered significant at *P*<0.05 and were considered a tendency when 0.05<*P*<0.10. All values are reported as LS means ± standard error of the mean (SEM).

## Results

We have previously reported that pre-partum infusion of serotonin to the dairy cows studied in this trial did not have any effects on feed intake, milk yield, body weight, or health parameters during the infusion period [21]. Of the 24 cows enrolled in our study, we have reported that two H CON, two H 5-HTP, three J CON, and two J 5-HTP cows were treated for ketosis. One H CON, three H 5-HTP, four J CON, and one J 5-HTP cow had mastitis. One H 5-HTP cow had a retained placenta. Finally, two H CON, two J CON, and one J 5-HTP cow required intravenous calcium administration post-partum due to clinical symptoms of hypocalcemia.

### Serotonin content was elevated in the livers of cows infused with 5-HTP

**Fig 1:**
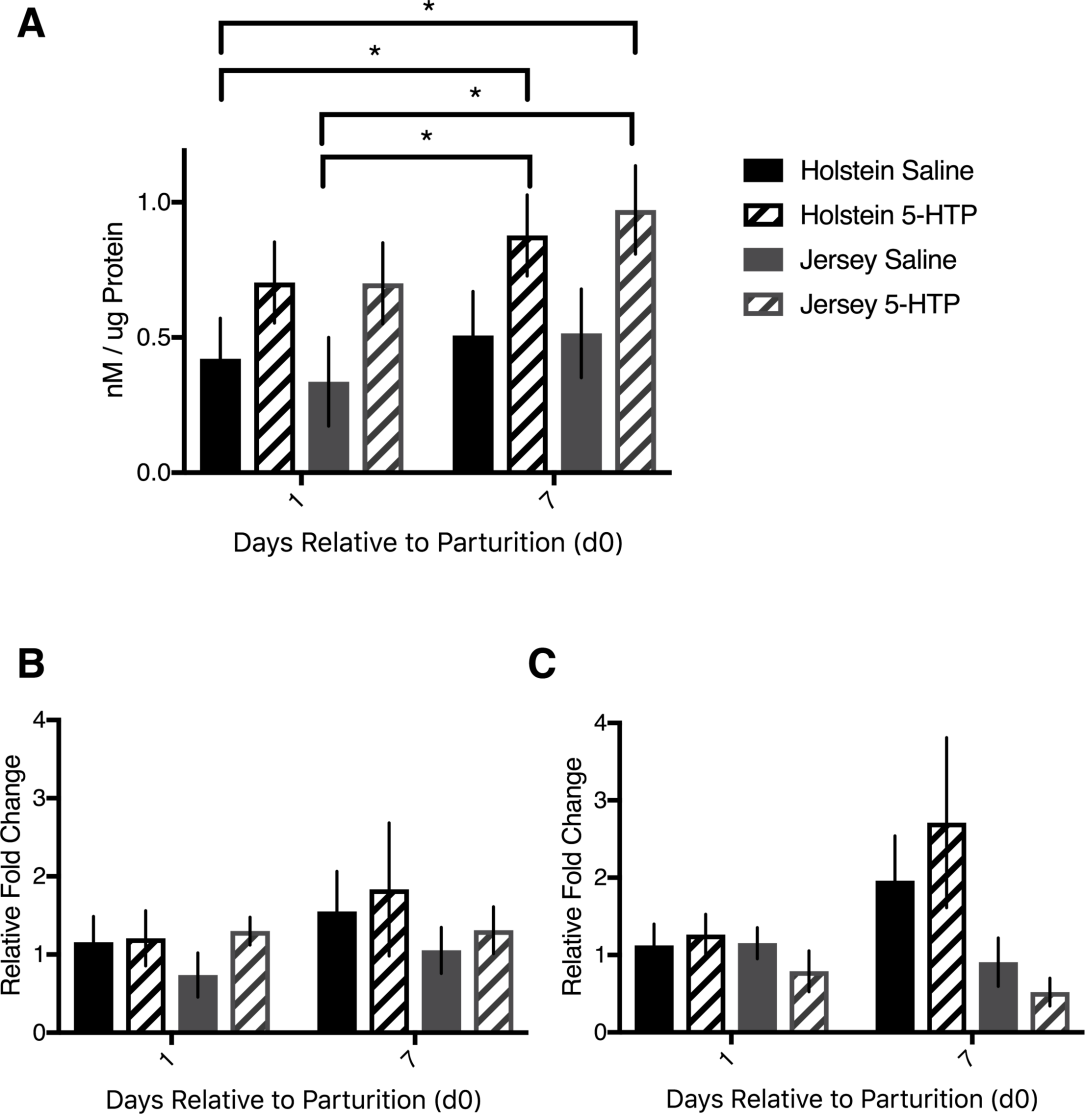
Hepatic serotonin content and *TPH1* and *SERT* mRNA abundance of multiparous Holstein cows and multiparous Jersey cows administered pre-partum daily I.V. infusions of 1 liter of saline or 1 liter of 1.0 mg/kg bodyweight of 5-hyroxy-L-tryptophan (5-HTP) reconstituted in saline. Final treatment groups were saline-infused Holsteins (*n* = 6), 5-HTP infused Holsteins (*n* = 6), saline-infused Jerseys (*n* = 6), and 5-HTP infused Jerseys (*n* = 6). (A) Liver serotonin content corrected to ug of protein (B) mRNA abundance of tryptophan hydroxylase 1 (*TPH1*) and (C) mRNA abundance of serotonin reuptake transporter (*SERT*). Stars indicate statistical difference between group means (* = 0.05<*P*<0.01). All values are reported as LS means ± SEM.

While we established that infusion of 5-HTP to both Holstein and Jersey cows elevated circulating serotonin concentrations [21], we wanted to determine whether serotonin content was elevated in the liver. Cows infused with 5-HTP had increased hepatic serotonin content compared to saline-infused cows (*P* = 0.006). All cows tended to have elevated serotonin content on d7 post-partum relative to d1 post-partum (*P* = 0.07). In particular, on d7, J 5-HTP tended to have elevated hepatic serotonin content over J CON (*P* = 0.06; Fig 1A). In order to establish if the increased serotonin content was due to elevated hepatic production, we measured the mRNA expression of the rate-limiting enzyme in serotonin synthesis, *TPH1*. The expression of *TPH1* did not differ across breeds or across treatments (*P*>0.05; Fig 1B). The serotonin reuptake transporter (*SERT*) mRNA abundance was elevated on d7 post-partum in Holstein cows (*P* = 0.02), but there was no effect of treatment (*P* = 0.32) or DRP by treatment interaction (*P* = 0.77). There were no differences in *SERT* mRNA abundance within the Jersey cows (*P*>0.05), but Holstein cows had elevated *SERT* mRNA abundance compared to Jersey cows (*P* = 0.03), particularly on d7 post-partum, as indicated by the breed by DRP interaction (*P* = 0.004; Fig 1C).

### Serotonin receptors 2A and 2B, but not 1D or 7, mRNA was more abundant in the livers of cows infused with 5-HTP

**Fig 2:**
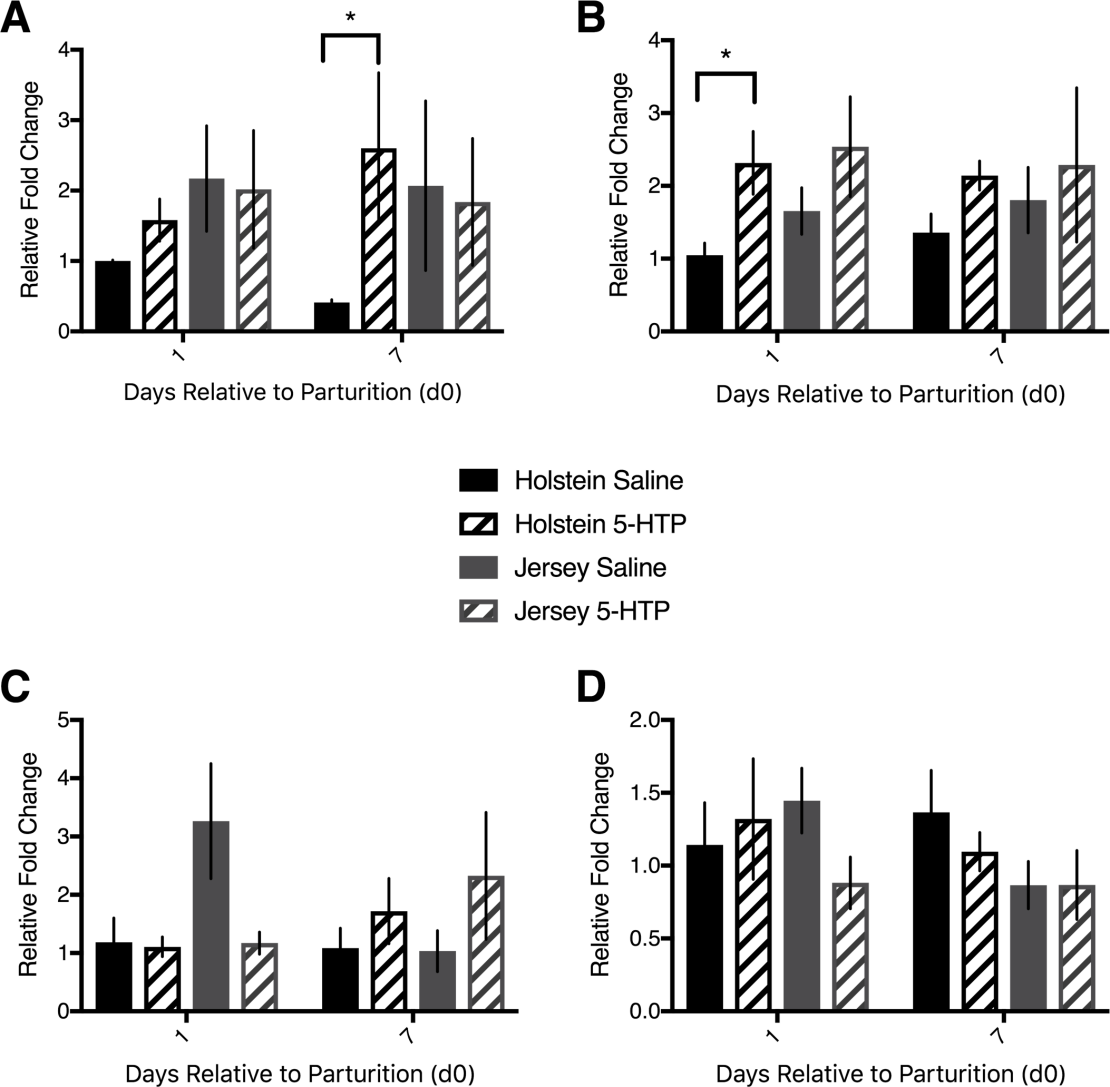
**Hepatic mRNA abundance of serotonin receptors *1D*, *2A*, *2B*, and *7* of multiparous Holstein cows and multiparous Jersey cows administered pre-partum daily I.V. infusions of 1 liter of saline or 1 liter of 1.0 mg/kg bodyweight of 5-hyroxy-L-tryptophan (5-HTP) reconstituted in saline.** Final treatment groups were saline-infused Holsteins (*n* = 6), 5-HTP infused Holsteins (*n* = 6), saline-infused Jerseys (*n* = 6), and 5-HTP infused Jerseys (*n* = 6). mRNA abundance of various serotonin receptors (*5HTR*s) (A) *5HTR2A* (B) *5HTR2B* (C) *5HTR1D* and (D) *5HTR7*. Stars indicate statistical difference between group means (* = 0.05<*P*<0.01). All values are reported as LS means ± SEM.

Given that serotonin content was elevated in the livers of cows infused with 5-HTP, it was pertinent to determine the activity of major serotonin receptors. mRNA abundance of *5HTR2A* was elevated in H 5-HTP over H CON cows overall (*P* = 0.05). There was no difference in mRNA abundance between d1 and d7 (*P* = 0.63) nor was there an interaction of 5-HTP treatment with DRP (*P* = 0.17) in Holstein cows. On d7 specifically, H 5-HTP cows had a nearly 3-fold increase in mRNA abundance of *5HTR2A* over H CON cows (*P* = 0.03). By contrast, there was no effect of treatment (*P* = 0.80), DRP (*P* = 0.78), or interaction between treatment and DRP (*P* = 0.98) in Jersey cows with respect to *5HTR2A* mRNA abundance. Comparison of Holstein and Jersey cows using H CON d1 samples as the internal control revealed no effect of breed (*P* = 0.32), treatment (*P* = 0.33), or DRP (*P* = 0.95) on either d1 or d7 post-partum (Fig 2A).

Holstein cows infused with 5-HTP had elevated *5HTR2B* mRNA abundance in their liver relative to H CON cows (*P* = 0.009). There was no difference between mRNA abundance on d1 and d7 (*P* = 0.68) or an interaction of day and treatment (*P* = 0.32). Jersey cows infused with 5-HTP tended to have greater *5HTR2B* mRNA abundance (*P* = 0.10) than J CON cows, with no effect of DRP (*P* = 0.24) or interaction of treatment and DRP (*P* = 0.26). When comparing Holstein cows and Jersey cows, using H CON cows d1 biopsy as the internal control, Jersey cows tended to have increased *5HTR2B* mRNA abundance compared to Holstein cows (*P* = 0.10) and there was an overall effect of treatment across all cows (*P* = 0.03; Fig 2B).

There were no effects of 5-HTP infusion, DRP, or any interaction within or between Holstein or Jersey cows with respect to *5HTR1D* hepatic mRNA abundance, with one exception. Within Jersey cows, there was an interaction of treatment and DRP (*P* = 0.05), with J CON having elevated *5HTR1D* mRNA abundance over J 5-HTP on d1, but not on d7 (Fig 2C). There were no changes across treatments, breeds, or DRP with respect to *5HTR7* hepatic mRNA abundance (*P*>0.05; Fig 2D).

### Markers of cell turnover CCND1, CDK4, PCNA, CASP3 were similar in mRNA abundance across treatments, but not breed

**Fig 3:**
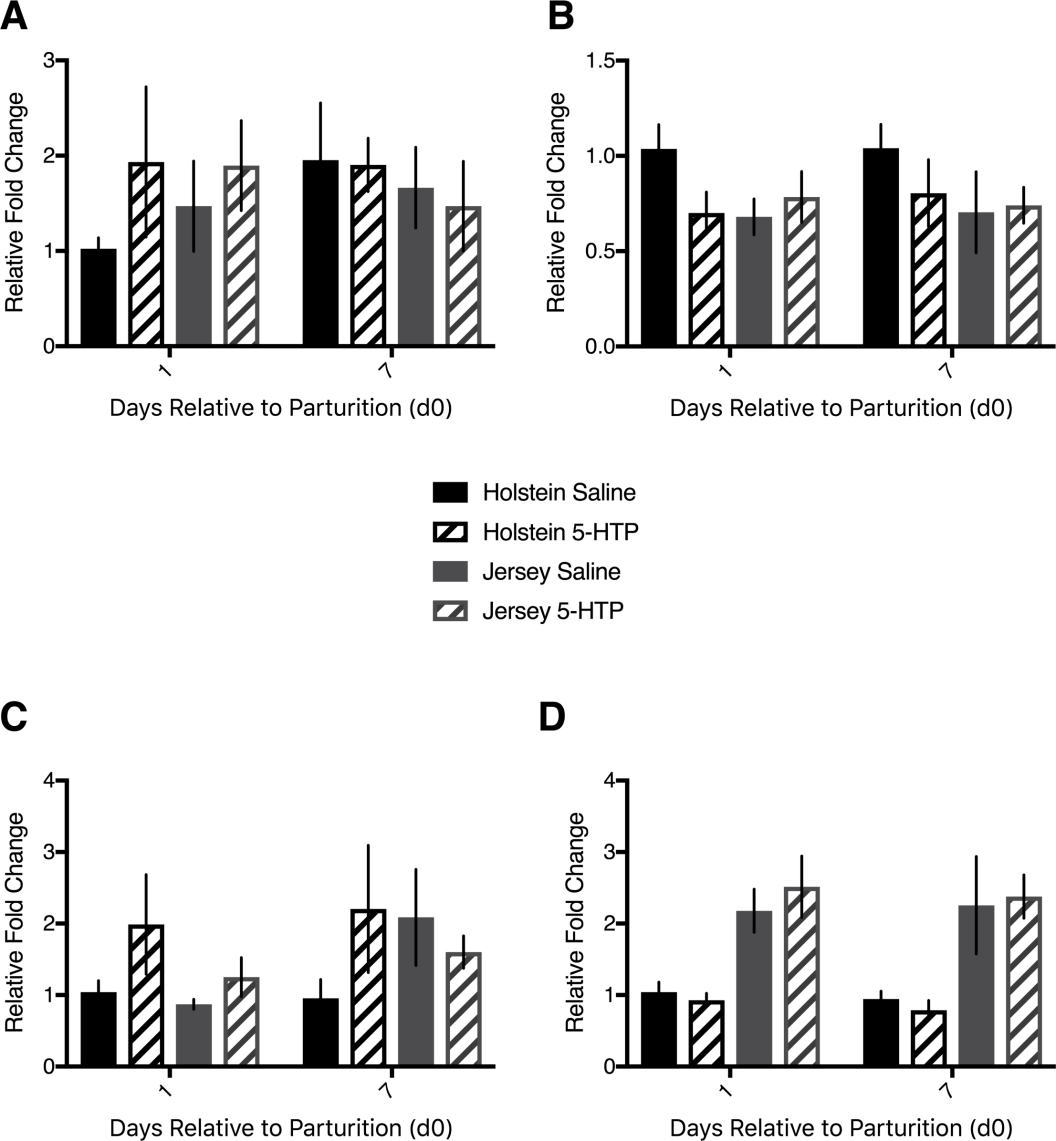
Hepatic mRNA abundance of *CCND1*, *CDK4*, *PCNA*, and *CASP3* in multiparous Holstein cows and multiparous Jersey cows administered pre-partum daily I.V. infusions of 1 liter of saline or 1 liter of 1.0 mg/kg bodyweight of 5-hyroxy-L-tryptophan (5-HTP) reconstituted in saline. Final treatment groups were saline-infused Holsteins (*n* = 6), 5-HTP infused Holsteins (*n* = 6), saline-infused Jerseys (*n* = 6), and 5-HTP infused Jerseys (*n* = 6). mRNA abundance of (A) cyclin D1 (*CCND1*) (B) cyclin-dependent kinase 4 (*CDK4*) (C) proliferating cell nuclear antigen (*PCNA*) and (D) caspase-3 (*CASP3*). All values are reported as LS means ± SEM.

The serotonin receptor 2B has clear associations with cell proliferation in the liver. As such, we measured classical markers of cell turnover at the mRNA level. There was no difference in Cyclin D1 (*CCND1*) mRNA abundance within Holsteins alone (P>0.05), Jerseys alone (P>0.05), nor were there any differences between the breeds (P>0.05; Fig 3A). Cyclin-dependent kinase 4 (*CDK4*) mRNA abundance was similar between Holstein cows, with H CON tending to have elevated abundance over H 5-HTP (*P* = 0.09), but no effect of DRP (*P* = 0.36) or interaction of treatment with DRP (*P* = 0.93). Within Jersey cows, there was no effect of treatment, DRP, or the interaction (P>0.05) with respect to *CDK4* hepatic mRNA abundance. Similarly, there was no difference in mRNA abundance between the two breeds (*P* = 0.15; Fig 3B). Proliferating cell nuclear antigen (*PCNA*) mRNA tended to be more abundant in H 5-HTP compared to H CON cow liver tissue (*P* = 0.09), with no effect of DRP (*P* = 0.90) or interaction between DRP and 5-HTP treatment (*P* = 0.98). Jersey cows tended to have elevated *PCNA* mRNA abundance on d7 compared to d1 postpartum (*P* = 0.06), with no effect of treatment (*P* = 0.88) or interaction between treatment and DRP (*P* = 0.26). Comparison of the breeds revealed no differences in *PCNA* mRNA abundance (*P*>0.05; Fig 3C). Apoptotic cell marker caspase-3 (*CASP3*) was not differentially expressed between treatments or across time in Holstein cows or Jersey cows separately (*P*>0.05). Interestingly, Jersey cows had increased mRNA abundance of *CASP3* relative to Holstein cows on both d1 and d7 (*P* = 0.0001; Fig 3D).

### Holstein cows infused with 5-HTP had more Ki67-positive hepatocytes than saline-infused cows on d1 post-partum, with similar changes in CASP3 protein expression

**Fig 4:**
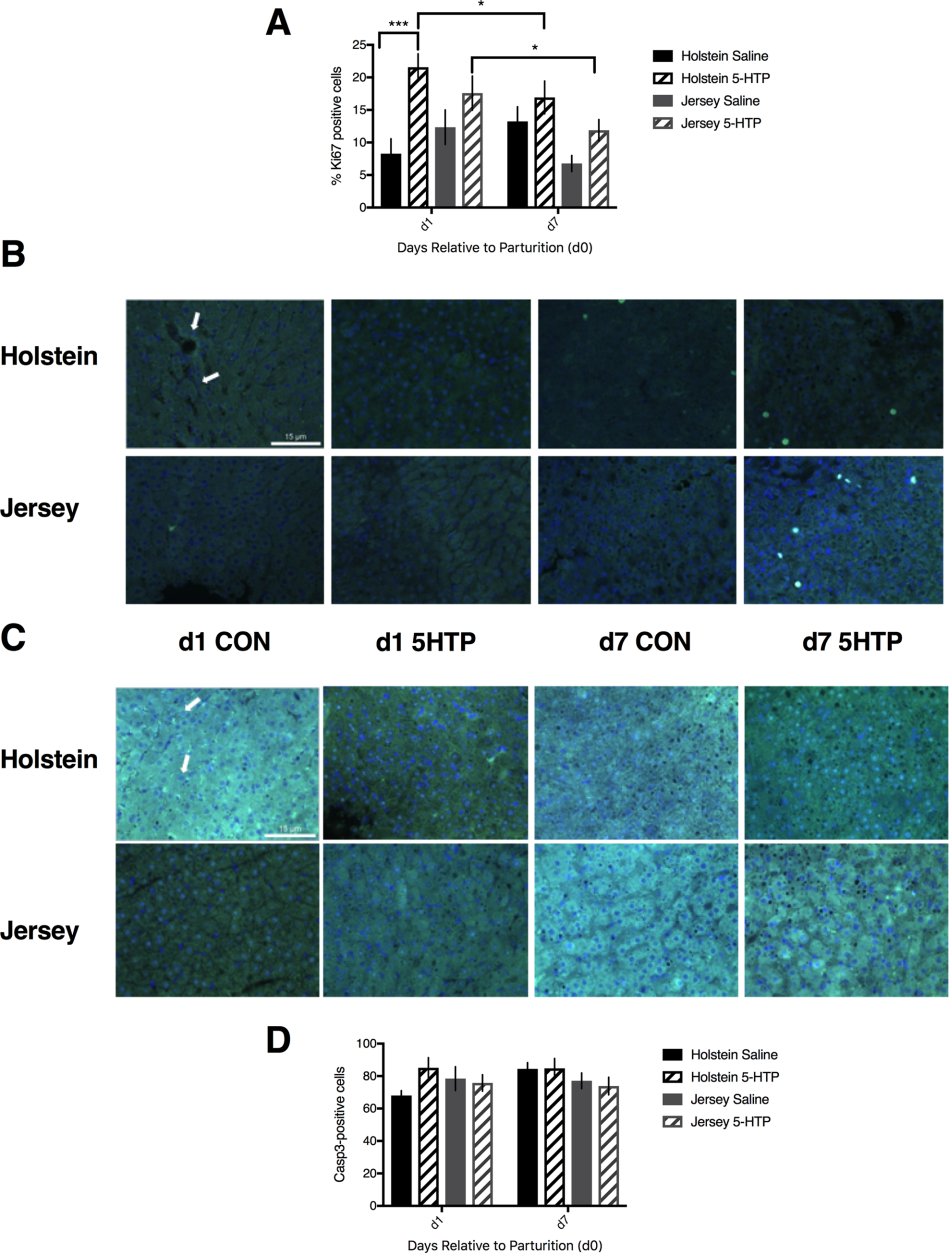
Immunofluorescent images and counts of cells positive for Ki67 and CASP3 in multiparous Holstein cows and multiparous Jersey cows administered pre-partum daily I.V. infusions of 1 liter of saline or 1 liter of 1.0 mg/kg bodyweight of 5-hyroxy-L-tryptophan (5-HTP) reconstituted in saline. Final treatment groups were saline-infused Holsteins (*n* = 6), 5-HTP infused Holsteins (*n* = 6), saline-infused Jerseys (*n* = 6), and 5-HTP infused Jerseys (*n* = 6). (A) Percent of cells positive for Ki67 stain of all counted cells in representative images (*n* = 3) from each treatment group. (B) Immunofluorescent images of Ki67-positive cells. The scale bar indicates 15 microns. The arrow indicates an example of a cell considered positive for Ki67. d1 and d7 are days 1 and 7 post-partum, respectively. CON and 5-HTP represent saline-infused and 5-HTP infused groups, respectively. (C) Immunofluorescent images of CASP3-positive cells. The scale bar indicates 15 microns. The arrow indicates an example of a cell considered positive for CASP3. (D) Count of cells positive for CASP3 in representative images (*n* = 3) from each treatment group. Stars indicate statistical difference between group means (* = 0.05<*P*<0.01; *** = 0.001<*P*<0.0001). All values are reported as LS means ± SEM.

Holstein cows infused with 5-HTP had a greater number of proliferating cells, as indicated by Ki67 staining, in their livers than H CON cows on d1, but not d7 post-partum, as indicated by the treatment by DRP interaction (*P* = 0.03). There was no effect of treatment alone (*P* = 0.11), nor any effect of DRP alone (*P* = 0.29) in Holstein cows. Jersey cows had more Ki67-positive cells on d1 compared to d7 post-partum (*P* = 0.02), but there was no effect of treatment (*P* = 0.15) or interaction of treatment and DRP (*P* = 0.73). In the model comparing the breeds, there was an overall effect of treatment (*P* = 0.03), as well as a DRP by breed interaction (*P* = 0.03), with both breeds having more Ki67-positive cells on d1 versus d7 post-partum (Fig 4A and 4B).

In order to examine cell apoptosis, we used immunofluorescence to measure Caspase 3 (CASP3) positive cells. Within Holstein cows, there was increased CASP3 detected in H 5-HTP cows compared to H CON cows on d1 (*P* = 0.04), but not d7, post-partum. There was no effect of treatment (*P* = 0.15) or DRP (*P* = 0.12). There was no change in the number of CASP3-positive cells among Jersey cows (P>0.05). A comparison of the breeds revealed no difference between Holstein and Jersey cows (P>0.05; Fig 4C and 4D).

### mRNA abundance of genes associated with gluconeogenesis and beta-oxidation were unresponsive to 5-HTP infusion, with few exceptions

**Fig 5:**
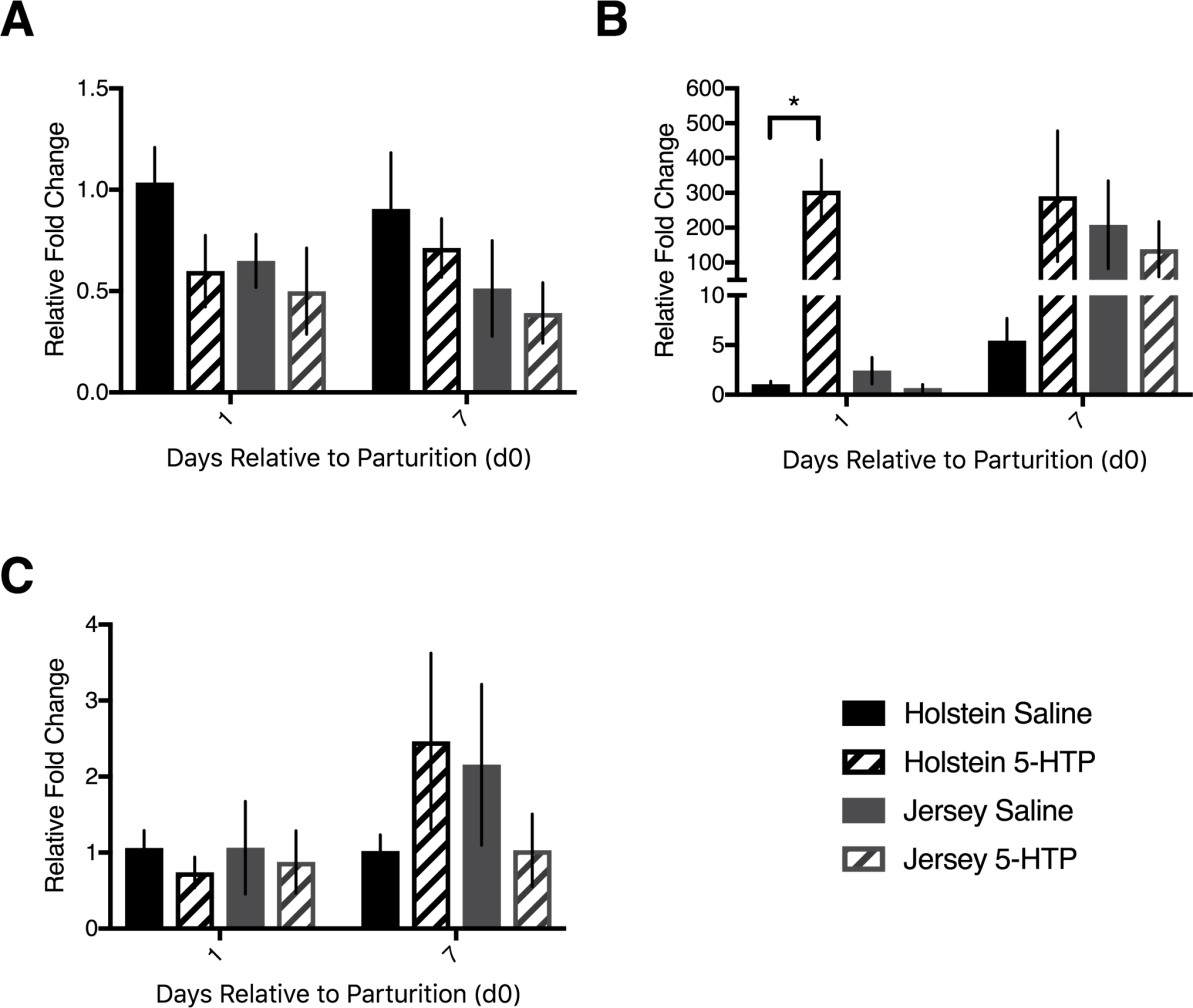
mRNA abundance of gluconeogenic (*PEPCK2*, *PC*, *G6P*) genes in multiparous Holstein cows and multiparous Jersey cows administered pre-partum daily I.V. infusions of 1 liter of saline or 1 liter of 1.0 mg/kg bodyweight of 5-hyroxy-L-tryptophan (5-HTP) reconstituted in saline. Final treatment groups were saline-infused Holsteins (*n* = 6), 5-HTP infused Holsteins (*n* = 6), saline-infused Jerseys (*n* = 6), and 5-HTP infused Jerseys (*n* = 6). mRNA abundance of (A) phosphoenolpyruvate carboxykinase 2, mitochondrial (*PEPCK2*), (B) pyruvate carboxylase (*PC*), (C) glucose 6 phosphatase (*G6P*). d1 and d7 are days 1 and 7 post-partum, respectively. CON and 5-HTP represent saline-infused and 5-HTP infused groups, respectively. Stars indicate statistical difference between group means (* = 0.05<*P*<0.01; *** = 0.001<*P*<0.0001). All values are reported as LS means ± SEM.

Evaluation of gluconeogenic enzymes was performed in the liver samples obtained on d1 and d7 post-partum. Holstein cows had greater expression of *PEPCK2* compared to Jersey cows on both d1 and d7 post-partum, irrespective of treatment (*P* = 0.05). While there was no effect of treatment or day within Holstein cows (*P*>0.05), expression of *PEPCK2* on d1 post-partum tended to be elevated in Jerseys compared to d7 post-partum (*P* = 0.06; Fig 5A). Pyruvate carboxylase mRNA expression in the liver was affected by the interaction of day with breed (*P* = 0.04) and tended to be affected by the interaction of treatment with breed (*P* = 0.08). Within Holstein cows alone, *PC* expression was elevated in H 5-HTP cows compared to H CON cows both on d1 and d7 post-partum, although more so on d1 post-partum (*P* = 0.01). Within Jersey cows, there was no effect of either day or treatment, although on d7 post-partum there was numerically greater expression of *PC* than on d1 post-partum (Fig 5B). There were no changes in the expression pattern of *G6P* (*P*>0.05; Fig 5C)

### Metabolites BHBA, glucose, insulin, glucagon, NEFA, and PUN were largely unaffected by 5-HTP infusion

In order to establish if there were larger metabolic effects of 5-HTP infusion, we analyzed various blood markers indicative of transition dairy cow health. Overall, we report that 5-HTP infusion had minimal effect on these parameters, as outlined in S1 Table, with several exceptions. Briefly, BHBA concentrations were affected by the interaction of treatment and DRP (*P* = 0.02), with J CON displaying elevated BHBA relative to all other groups on approximately d6 to d10 post-partum. When each breed was analyzed separately, represented in S2 Table, there was no effect of treatment within either breed, although DRP did have an effect on BHBA levels in Holstein cows only (*P* = 0.008). When each treatment group was analyzed separately, represented in S3 Table, there was an effect of DRP (*P* = 0.03) and a breed by DRP interaction (*P* = 0.003) within all cows infused with 5-HTP on BHBA concentrations. The separation between breeds is most evident on d9 through d13, when J 5-HTP have elevated BHBA relative to H 5-HTP (S1A Fig).

Glucose levels had their characteristic peak at parturition and decline post-partum in all cows (*P*<0.0001). Additionally, Holstein cows had overall elevated glucose levels relative to Jersey cows (*P* = 0.003). While DRP affected glucose levels in each breed (*P*<0.0001 for each), there was no effect of treatment or interaction between DRP and treatment in either breed (P>0.05). Within both saline (*P* = 0.03) and 5-HTP-treated (*P* = 0.04) cows, Holsteins had elevated glucose levels compared to Jersey cows (S1B Fig).

While there were changes in plasma insulin levels dependent on DRP (*P*<0.0001), there were no other distinguishable breed or treatment effects with respect to insulin levels (S1C Fig). Plasma glucagon levels were more dynamic, however, with H CON cows’ plasma glucagon levels increasing with increasing with time post-partum, as evidenced by the overall breed by DRP (*P* = 0.05) and treatment by breed by DRP interactions (*P* = 0.01). Within Holstein cows, H CON cows’ elevated glucagon was only a tendency (*P* = 0.07), while glucagon in Holstein cows was definitively affected by DRP (*P* = 0.002). This trend was also reflected when comparing the saline-infused cows, with a breed by DRP interaction (P = 0.02) as H CON cows had elevated glucagon levels over J CON cows (S1D Fig). Finally, non-esterified fatty acid levels changed over time (*P*<0.0001), but were not affected by treatment or breed (*P*>0.05; S1E Fig), while plasma urea nitrogen levels were not affected by DRP, treatment, or breed (*P*>0.05; S1F Fig).

## Discussion

Serotonin has been established as a potent regulator of energy balance [2, 7, 8, 16]. Dairy cows going through the transition from pregnancy to lactation undergo large metabolic changes, due to the rapid and significant demand for milk synthesis. An inability of the cow to adapt metabolically can result in a variety of transition-related disorders. We have recently established that serotonin may be an effective therapeutic target for one of these diseases: hypocalcemia [21]. Given that hypocalcemia typically precedes energy-related conditions such as ketosis or fatty liver, we were interested in whether manipulation of the serotonergic axis also affected energy homeostasis. The objective of this study was to evaluate whether administration of 5-HTP, the immediate precursor to serotonin, could improve energy homeostasis or local liver dynamics in the early lactation dairy cow.

Evaluation of serotonin content in the liver revealed a clear response to 5-HTP infusion, as all cows infused with 5-HTP had elevated serotonin content. As there were no changes in *TPH1* expression in the liver, this increase is likely a result of increased serotonin uptake from the circulation resulting from the 5-HTP treatments. Given serotonin’s role as an autocrine-paracrine regulator of various organs during lactation [26], we set out to examine the effects of 5-HTP infusion on local liver dynamics. Serotonin has long been known to mediate hepatocyte proliferation. In primary cultures of rat hepatocytes, serotonin was shown to induce a dose-dependent increase in DNA synthesis in the presence of insulin and EGF [27]. The serotonin receptor 2 family has received much of the credit for the regulation of cell proliferation. In the present study, both Holstein and Jersey cows had increased mRNA abundance of *5HTR2B* following infusion of 5-HTP, although this response was more robust in Holstein cows, particularly on d1 post-partum. Holstein cows infused with 5-HTP also had elevated *5HTR2A* mRNA abundance over H CON on d7 post-partum, while Jersey cows did not. Serotonin expedites liver regeneration through the 2A and 2B receptors, effectively rescuing the age-associated decline in regeneration and survival following liver resection [13, 28]. Mice with sufficient serotonin that are exposed to acetaminophen have a lower mortality rate than those mice with insufficient serotonin; this is thought to be in part because of elevated hepatocyte proliferation and activation of the *5HTR2B* receptor preventing toxicity [29]. In addition, the blockade of serotonin 2 receptors can arrest liver regeneration when administered close to the G1/S transition point of the cell cycle [30] and *5HTR2B* may be a therapeutic target in hepatocellular cancer due to its association with high levels of proliferation and tumor growth in human hepatocellular cancer cell lines [31, 32]. Given that *5HTR1D* was elevated in the livers of wethers intravenously injected with serotonin [20] and that the *5HTR7* receptor has also been implicated in hepatic regeneration [33], we also examined mRNA abundance of these receptors. Neither was responsive to 5-HTP infusion in our dairy cows, aside from elevated *5HTR1D* expression in J CON over J 5-HTP cows on d1 post-partum. Given the change in *5HTR1D* was not accompanied by any biologically relevant markers measured in the present study, the potential role of this receptor has to be further examined and is likely not associated with proliferation at this particular dose of 5-HTP in dairy cows.

Increased hepatocyte proliferation is necessary during lactation in order to accommodate the demand for glucose, among other factors, from the mammary gland. While 34% of total glucose turnover is oxidized to carbon dioxide on day 30 pre-partum, turnover decreases to only 8 to 9% by day 7 of lactation [34]. These increased energy requirements put enormous demands on the liver. Indeed, liver mass is increased in dairy cows as the lactation progresses, largely due to increased cell size and cell number [35, 36]. Liver size is related to physiological workload, and the liver will increase in size in order to meet physiological demands [37]. Serotonin administration has also been shown to increase liver weight, indicating a positive effect of serotonin on liver growth [38]. Given that progression through G1 and the G1/S transition in mammalian cells is regulated by cyclin D1 and CDK4, among other factors, it was pertinent to examine various cell cycle markers in our hepatic samples. While there were no alterations in *CCND1* or *CDK4* mRNA abundance, H 5-HTP cows tended to have elevated *PCNA* mRNA abundance over H CON cows. Importantly, at the level of the protein, the trend established in the *PCNA* mRNA pattern was reinforced by the increased number of Ki67-positive cells in H 5-HTP cows compared to H CON cows on d1 post-partum. This pattern was less pronounced on d7 post-partum and was not evident in Jersey cows. As such, not only do Holsteins and Jerseys respond differently to 5-HTP infusion, but the immediate onset of lactation also demands more rapid proliferation of the liver compared to even one week post-partum. Given that cyclin D1 is the marker for cell cycle progression in hepatocytes [39], our hepatic samples taken after the initiation of lactation may have been too late to detect changes in cyclin D1 expression at the level of the mRNA. Additionally, not only is PCNA an essential eukaryotic DNA replication factor, but it also plays a role in DNA repair [40]. As such, changes in PCNA might reflect more than simply increased proliferation, but also increased cell viability through DNA fidelity. At present, the role of 5-HTP in mediating DNA repair is simply speculation, but warrants further examination in the future.

In addition to elevated cellular proliferation, lactation is characterized by increased cell turnover [41]. For example, mitochondrial DNA in the liver is tightly correlated with mitochondrial DNA in the peripheral blood during early, but not late, lactation [42], reflecting the large amount of tissue biogenesis occurring during early lactation. Markers of cell apoptosis, such as single-stranded DNA, caspase-3 activity, and nuclear chromatin, were increased the week of parturition and at 3 weeks post-partum compared to 3 weeks pre-partum in dairy cows and in cows with fatty infiltration of the liver [43, 44]. There was increased *CASP3* transcript in the present study on d1 post-partum in Holstein cows compared to d7, and immunofluorescence staining revealed increased CASP3 protein in H 5-HTP cows compared to H CON cows on d1 of lactation. Given that there were no associated changes in circulating NEFA concentrations or increased diagnosis of fatty liver in 5-HTP cows, we propose that elevated CASP3 protein levels are instead indicative of increased cell turnover and tissue remodeling necessary during the onset of lactation on d1 post-partum in Holstein cows. This is supported by the significant increase in cell proliferation that was also observed by Ki67 staining. Holstein cows treated with 5-HTP had reduced feed intake for two days pre-partum compared to H CON, while J 5-HTP had reduced feed intake for four days pre-partum compared to J CON [21]. As such, increased CASP3 protein in H 5-HTP on d1 could be reflecting a detrimental shift in the balance towards hepatic apoptosis pre-partum. However, given that there were no differences in milk production aside from J CON producing less than J 5-HTP on the day of calving [21], it is unlikely that there was any detrimental shift towards hepatic apoptosis that would negatively affect feed intake or milk production.

Given the changes in hepatocyte turnover and proliferation, it was pertinent to measure various endpoints of glucose metabolism in the liver. Previous work has shown that *PC* mRNA increased 6-fold at one day post-partum and that cytosolic, but not mitochondrial, *PEPCK* increased 2.5-fold at two weeks post-partum compared to one day post-partum in transition dairy cows [45]. Pyruvate carboxylase synthesizes oxaloacetate (OAA) from pyruvate. Gluconeogenesis, insulin secretion, and tricarboxylic acid cycle activity all require OAA, so PC acts as a crucial checkpoint in ensuring metabolic balance. PEPCK can also drain the OAA pool in the process of committing carbons to gluconeogenesis. As such, the ratio of PC to PEPCK is crucial to hepatic metabolic balance in the transition dairy cow. If PC activity is not sufficient to balance PEPCK activity, the OAA pool could be depleted and shift liver metabolism away from gluconeogenesis [45]. mRNA abundance of *PC* was dramatically upregulated in Holstein transition dairy cows administered 5-HTP, without any shift in *PEPCK2* expression. Caution must be taken in interpreting these results, as *PC* underwent a dramatic upregulation of approximately 200-fold change in response to 5-HTP, which certainly warrants further investigation. The increase in *PC*, but not *PEPCK2*, with 5-HTP administration suggests that OAA precursors were sufficient to support gluconeogenesis, although *PEPCK1* (cytosolic) mRNA expression was not measured and could have responded to 5-HTP differently than *PEPCK2*. Notably, there were no changes in the mRNA expression of *G6P* as a result of treatment with 5-HTP or breed. Previously, gut-derived serotonin was shown to increase activity of G6P in mice, which hydrolyzes glucose-6-phosphate to result in free glucose as the final step of gluconeogenesis [9]. Interestingly, intravenous infusion of extremely high doses of glucose to dairy cows affected G6P activity, but not mRNA abundance, indicating post-transcriptional regulation of this enzyme is ultimately responsible for circulating glucose status [46]. When comparing data acquired from rodents compared to that from dairy cows, it is essential to distinguish between ruminants and non-ruminants with respect to gluconeogenesis. This distinction arises from cows’ high capacity for gluconeogenesis from propionate due to the demands for glucose in milk [47]. Collectively, the mRNA adaptations seen in this study support the possibility of increased gluconeogenesis due to increased *PC*, but not *PEPCK2*, abundance in the livers of early lactation Holstein dairy cows infused with 5-HTP, although no changes in glucose were noted in *G6P* expression. Further work is necessary to confirm the activity of these mRNA markers at the protein and enzymatic activity levels in the transition dairy cow.

The changes evident in the local dynamics of proliferation and cell turnover in the liver were not reflected at the level of the circulation, with a few exceptions. Interestingly, BHBA concentrations were elevated in J CON cows relative to all other treatment groups in the window of d6 to d10 post-partum. Concentrations of BHBA are the main tool used in the diagnosis of both subclinical (BHBA > 1.2 mmol/L) and clinical (BHBA > 3.0 mmol/L) ketosis. While clinical ketosis is certainly an issue in modern dairy herds, the detection of subclinical ketosis is arguably more important, as visible symptoms are difficult to detect and a greater proportion of the herd (15 to 60% as compared to 2 to 15%) is likely to be affected [48]. Elevated plasma BHBA concentrations are associated with hepatic damage, oxidative stress, reproductive delays, reduced milk production, and a variety of adverse health events [1, 49, 50]. The tendency towards reduced BHBA levels in Jersey cows infused with 5-HTP must be interpreted with caution, particularly as J 5-HTP cows in this time window also had reduced circulating total calcium concentrations [21]. Previously, work from Martinez and coworkers has shown that cows with subclinical hypocalcemia had greater concentrations of BHBA, although that study was performed in Holstein cows [51]. Clearly, the relationship between these metabolites during the transition period warrants closer examination with more frequent sampling and experimental control of feeding time, for example.

Aside from reduced BHBA levels in a narrow window post-partum, however, there were few changes in circulating glucose, insulin, glucagon, NEFA, or PUN parameters in the circulation. Energy-related metabolites must be tightly regulated during the transition period in order to provide adequate precursors for milk synthesis while maintaining the health of the cow. The cows in this study had altered calcium homeostasis as a result of 5-HTP infusion [21]. It is therefore important that there were no detrimental effects of 5-HTP infusion on energy status. Notably, previous work in our lab aimed at determining the optimal dose of 5-HTP that was performed in late-lactation, non-pregnant dairy cows showed acute changes in several of these parameters at both 1.0 mg/kg and 1.5 mg/kg within two hours post-infusion. For example: plasma glucose was elevated, plasma insulin was decreased, NEFA concentrations were increased, and BHBA was decreased [22]. During the transition period, these metabolites are much more tightly regulated due to the onset and metabolic demands of lactation. It is possible that administration of a higher dose of 5-HTP, such as the 1.5 mg/kg dose used in the previous study, would elicit a more robust response in transition period dairy cows.

Additionally, blood samples in the current study were not collected at a consistent time relative to feeding, as the study was initially designed to examine calcium homeostasis [21]. As such, profiles of metabolites should be considered with relative caution. One study found that periprandial concentrations of blood metabolites in Holstein cows were affected for the first 3 months post-partum by when samples were taken relative to feeding. Specifically, urea, BHBA, and insulin showed relatively immediate increases post-prandially, while NEFA and glucose concentrations decreased sharply within the first 1 to 3 hours post-feeding. Aside from glucose, which recovered by 6 to 9 hours post-feeding, all other metabolites pertinent to this study remained relatively steady for the next 6 to 9 hours. The authors concluded that the most useful and opportune time to sample early-lactation dairy cows’ blood for the purpose of energy balance is before the morning feeding [52]. As the current study did not observe robust increases or decreases in any of the given metabolites, it is unlikely that there were periprandial effects of the time of blood sampling. On the other hand, the authors cannot rule out the potential that time of sampling relative to feeding affected any given metabolite. In future studies examining serotonin’s role in the regulation of energy balance, care should be taken to collect blood samples at a similar time relative to feeding. Given the potential benefits of a 1.0 mg/kg 5-HTP infusion during the transition period on calcium metabolism, particularly to Holstein cows, [21], it is encouraging to have experienced no adverse effects of 5-HTP infusion on energy metabolites. A larger study examining timing and dose of 5-HTP may reveal the benefits of 5-HTP infusion on energy status during the transition period. In addition, cows should be given more than one week to adapt to their new surroundings in future studies, as this adaptation period could affect important factors related to immunity and subsequent related metabolites.

Finally, several parameters in this study responded differently dependent on the breed of dairy cow. Notably, there was no statistically significant difference in the amount of time that Holstein cows were infused versus Jersey cows and all cows were managed similarly, including the same feed, at the same facility. As such, differences between the two breeds can comfortably be associated to inherent physiological differences in the Jersey cow and Holstein cow. For example, the mRNA expression of *CASP3* was elevated in Jersey cows compared to Holstein cows, although this was not reflected in the protein. Additionally, glucose levels were elevated in Holsteins compared to Jersey cows. One study showed similar glucose concentrations between the two breeds during late gestation [53]. By contrast, a different study demonstrated that cows on a Jersey farm had numerically lower concentrations of glucose in early lactation compared to cows from a Holstein farm, although the two farms were not compared statistically due to management differences [54]. Few studies have compared the incidence of ketosis between the two breeds, although it appears as if there are not significant differences between Holstein cows and Jersey cows [55]. Given that both insulin and glucagon were also affected by breed in this study (with insulin slightly elevated and glucagon slightly decreased in Jersey cows compared to Holstein cows), further work should carefully consider the breed of cow before making generalized statements about energy homeostasis. It is particularly important to consider breed due to the potential variations in diet, health status, site of blood collection, and milk yield that can differ between trials.

Infusion of 5-HTP to Holstein and Jersey dairy cows during the transition period increases serotonin content, alters the profile of the *5HTR2* family of receptors, and elevates markers of cellular proliferation and cell turnover in the liver on d1 and d7 post-partum. Specifically, Holstein cows infused with 5-HTP have increased *5HTR2A*, *5HTR2B*, and *PCNA* mRNA abundance, as well as increased numbers of CASP3 and Ki67-positive cells when compared to control cows. In addition, H 5-HTP cows have increased *PC*, but not *PEPCK2*, mRNA expression compared to H CON cows. Importantly, these changes are accompanied by only minor effects (reduced milk yield on d1 in J 5-HTP versus J CON cows, for example) on a variety of health-related parameters detectable in the circulation. In fact, our data reflects a trend towards decreased BHBA concentrations during a key window at 6 to 10 days post-partum in Jersey cows, with the important caveat that circulating calcium concentrations were also decreased in this given time window in Jersey cows. Given that the effects of serotonin are heavily dependent on dose and on timing, further studies should examine the comparative benefits of administering 5-HTP at a higher dose and potentially further into the lactation. Pre-partum infusions of 1.0 mg/kg 5-HTP alter local, hepatic serotonin dynamics towards increased cell proliferation necessary to meet the demands of lactation primarily in Holstein cows, with no adverse effects at the level of the circulation or adverse health outcomes.

## Supporting information

S1 Fig Profiles of various metabolites in the circulation of multiparous Holstein cows and multiparous Jersey cows administered pre-partum daily I.V. infusions of 1 liter of saline or 1 liter of 1.0 mg/kg bodyweight of 5-hyroxy-L-tryptophan (5-HTP) reconstituted in saline. Final treatment groups were saline-infused Holsteins (*n* = 6), 5-HTP infused Holsteins (*n* = 6), saline-infused Jerseys (*n* = 6), and 5-HTP infused Jerseys (*n* = 6). On average, Holstein cows were infused for 5.67 ± 0.78 days and Jersey cows were infused for 8.67 ± 1.53 days. Profiles of (A) beta-hydroxybutyrate (BHBA) (B) glucose (C) insulin (D) glucagon (E) non-esterified fatty acid (NEFA) and (F) plasma urea nitrogen (PUN) levels in either plasma or serum for either 7 or 14 days and on d30 post-partum. All values are reported as LS means ± SEM.
(TIFF)

S1 Table Main effects and interactions of treatment, day relative to parturition, and breed on various metabolites post-partum. (DOCX)

S2 Table Main effects and interactions of treatment and day relative to parturition within holstein cows or jersey cows on various metabolites post-partum. (DOCX)

S3 Table Main effects and interactions of breed and day relative to parturition within saline- or 5-HTP-infused cows on various metabolites post-partum. (DOCX)

## References

[1] McArt JAA, Nydam DV, Oetzel GR. Epidemiology of subclinical ketosis in early lactation dairy cattle. J Dairy Sci. 2012;95: 5056–5066. doi: 10.3168/jds.2012-5443 22916909 10.3168/jds.2012-5443

[2] Donovan MH, Tecott LH. Serotonin and the regulation of mammalian energy balance. Front Neurosci. 2013;7: 36 doi: 10.3389/fnins.2013.00036 23543912 10.3389/fnins.2013.00036 PMC3608917

[3] Laporta J, Keil KP, Vezina CM, Hernandez LL. Peripheral serotonin regulates maternal calcium trafficking in mammary epithelial cells during lactation in mice. PLoS One 2014;9: e110190 doi: 10.1371/journal.pone.0110190 25299122 10.1371/journal.pone.0110190 PMC4192539

[4] Mann JJ, McBride PA, Brown RP, Linnoila M, Leon AC, DeMeo M, et al Relationship between central and peripheral serotonin indexes in depressed and suicidal psychiatric inpatients. Arch Gen Psychiatry 1992;49: 442–446. 1376106 10.1001/archpsyc.1992.01820060022003

[5] Merens W, Willem Van der Does AJ, Spinhoven P. The effects of serotonin manipulations on emotional information processing and mood. J Affect Disord. 2007;103: 43–62. doi: 10.1016/j.jad.2007.01.032 17363069 10.1016/j.jad.2007.01.032

[6] Lam DD, Garfield AS, Marston OJ, Shaw J, Heisler LK. Brain serotonin system in the coordination of food intake and body weight. Pharmacol Biochem Behav. 2010;97: 84–91. doi: 10.1016/j.pbb.2010.09.003 20837046 10.1016/j.pbb.2010.09.003

[7] Oh CM, Park S, Kim H. Serotonin as a new therapeutic target for diabetes mellitus and obesity. Diabetes Metab. 2016;40: 89–98. 10.4093/dmj.2016.40.2.89 PMC4853228 27126880

[8] Oh CM, Namkung J, Go Y, Shong KE, Kim K, Kim H, et al Regulation of systemic energy homeostasis by serotonin in adipose tissues. Nat Commun. 2015;6: 6794 doi: 10.1038/ncomms7794 25864946 10.1038/ncomms7794 PMC4403443

[9] Sumara G, Sumara O, Kim JK, Karsenty G. Gut-derived serotonin in a multifunctional determinant to fasting adaptation. Cell Metab. 2012;16: 588–600. doi: 10.1016/j.cmet.2012.09.014 23085101 10.1016/j.cmet.2012.09.014 PMC3696514

[10] Kim H, Toyofuku Y, Lynn FC, Chak E, Uchida T, Mizukami H, et al Serotonin regulates pancreatic beta cell mass during pregnancy. Nat Med. 2010;16: 804–808. doi: 10.1038/nm.2173 20581837 10.1038/nm.2173 PMC2921604

[11] Ohara-Imaizumi M, Kim H, Yoshida M, Fujiwara T, Aoyagi K, Toyofuku Y, et al Serotonin regulates glucose-stimulated insulin secretion from pancreatic B cells during pregnancy. Proc Natl Acad Sci USA. 2013;110: 19420–19425. doi: 10.1073/pnas.1310953110 24218571 10.1073/pnas.1310953110 PMC3845121

[12] Kim HJ, Kim JH, Noh S, Hur HJ, Sung MJ, Hwang JT, et al Metabolomic analysis of livers and serum from high-fat diet induced obese mice. J Proteome Res 2011;10: 722–731. doi: 10.1021/pr100892r 21047143 10.1021/pr100892r

[13] Starlinger P, Assinger A, Haegele S, Wanek D, Zikeli S, Schauer D, et al Evidence for serotonin as a relevant inducer of liver regeneration after liver resection in humans. Hepatology 2014;60: 257–266. doi: 10.1002/hep.26950 24277679 10.1002/hep.26950

[14] Lesurtel M, Clavien PA. Platelet-derived serotonin: translational implications for liver regeneration. Hepatology 2014;60: 30–33. doi: 10.1002/hep.27067 24700245 10.1002/hep.27067

[15] Laporta J, Peters TL, Weaver SR, Merriman KE, Hernandez LL. Feeding 5-hydroxy-l-tryptophan during the transition from pregnancy to lactation increases calcium mobilization from bone in rats. Domest Anim Endocrinol. 2013;44: 176–184. doi: 10.1016/j.domaniend.2013.01.005 23433710 10.1016/j.domaniend.2013.01.005

[16] El-Merahbi R, Löfffler M, Mayer A, Sumara G 2015 The roles of peripheral serotonin in metabolic homeostasis. FEBS Lett. 2015;589: 1728–1734. doi: 10.1016/j.febslet.2015.05.054 26070423 10.1016/j.febslet.2015.05.054

[17] Aschenbach JR, Kristensen NB, Donkin SS, Hammon HM, Penner GB. Gluconeogenesis in dairy cows: the secret of making sweet milk from sour dough. IUBMB Life 2010;62: 869–877. doi: 10.1002/iub.400 21171012 10.1002/iub.400

[18] Adewuyi AA, Gruys E, van Eerdenburg FJ. Non esterified fatty acids (NEFA) in dairy cattle. A review. Vet Q. 2005;27: 117–126. doi: 10.1080/01652176.2005.9695192 16238111 10.1080/01652176.2005.9695192

[19] Laporta J, Hernandez LL. Serotonin receptor expression in dynamic in the liver during the transition period in Holstein dairy cows. Domest Anim Endocrinol. 2015a;51: 65–73. 25528206 10.1016/j.domaniend.2014.11.005

[20] Watanabe H, Saito R, Nakano T, Takahashi H, Takahashi Y, Sumiyoshi K, et al Effect of peripheral 5-HT on glucose and lipid metabolism in wether sheep. PLoS One 2014;9: e88058 doi: 10.1371/journal.pone.0088058 24505376 10.1371/journal.pone.0088058 PMC3913723

[21] Weaver SR, Prichard AP, Endres EL, Newhouse SA, Peters TL, Crump PM, et al Elevation of circulating serotonin improves calcium dynamics in the peripartum dairy cow. J Endocrinol. 2016a;230: 105–123. 27390301 10.1530/JOE-16-0038

[22] Laporta J, Moore SA, Weaver SR, Cronick CM, Olsen M, Prichard AP, et al Increasing serotonin concentrations alter calcium and energy metabolism in dairy cows. J Endocrinol. 2015b;226: 43–55. 26099356 10.1530/JOE-14-0693

[23] Karkalas J. An improved enzymic method for the determination of native and modified starch. J Sci Food Agr. 1985;36: 1019–1027.

[24] Vicari T, van den Borne JJGC, Gerrits WJJ, Zbindin Y, Blum JW. Postprandial blood hormone and metabolite concentrations influenced by feeding frequency and feeding level in veal calves. Domest Anim Endocrinol. 2008;34: 74–88. doi: 10.1016/j.domaniend.2006.11.002 17223005 10.1016/j.domaniend.2006.11.002

[25] Livak KJ, Schmittgen TD. Analysis of relative gene expression data using real-time quantitative PCR and the 2(-Delta Delta C(T)) method. Methods 2001;25: 402–408. doi: 10.1006/meth.2001.1262 11846609 10.1006/meth.2001.1262

[26] Weaver SR, Hernandez LL. Autocrine-paracrine regulation of the mammary gland. J Dairy Sci. 2016b;99: 842–853. 26299162 10.3168/jds.2015-9828

[27] Balasubramanian S, Paulose CS. Induction of DNA synthesis in primary cultures of rat hepatoyctes by serotonin: possible involvement of serotonin S2 receptor. Hepatology 1998;27: 62–66. doi: 10.1002/hep.510270111 9425918 10.1002/hep.510270111

[28] Lesurtel M, Graf R, Aleil B, Walther DJ, Tian Y, Jochum W, et al Platelet-derived serotonin mediates liver regeneration. Science 2006;312: 104–107. doi: 10.1126/science.1123842 16601191 10.1126/science.1123842

[29] Zhang J, Song S, Pang Q, Zhang R, Zhou L, Liu S, et al Serotonin deficiency exacerbates acetaminophen-induced liver toxicity in mice. Sci Rep. 2015;5: 8098 doi: 10.1038/srep08098 25631548 10.1038/srep08098 PMC4309973

[30] Papadimas GK, Tzirogiannis KN, Panoutsopoulos GI, Demonakou MD, Skaltsas SD, Hereti RI, et al Effect of serotonin receptor 2 blockage on liver regeneration after partial hepatectomy in the rat liver. Liver Int. 2006;26: 352–361. doi: 10.1111/j.1478-3231.2005.01230.x 16584399 10.1111/j.1478-3231.2005.01230.x

[31] Soll C, Jang JH, Riener MO, Moritz W, Wild PJ, Graf R, Clavien PA. Serotonin promotes tumor growth in human hepatocellular cancer. Hepatology 2010;51: 1244–1254. doi: 10.1002/hep.23441 20099302 10.1002/hep.23441

[32] Soll C, Riener MO, Oberkofler CE, Hellerbrand C, Wild PJ, DeOliveira ML, et al Expression of serotonin receptors in human hepatocellular cancer. Clin Cancer Res 2012;18: 5902–5910. doi: 10.1158/1078-0432.CCR-11-1813 23087410 10.1158/1078-0432.CCR-11-1813

[33] Tzirogiannis KN, Kourentzi KT, Zyga S, Papalimneou V, Tsironi M, Grypioti AD, et al 2014 Effect of 5-HT7 receptor blockade on liver regeneration after 60–70% partial hepatectomy. BMC Gastroenterol. 2014;14: 201 doi: 10.1186/s12876-014-0201-2 25433672 10.1186/s12876-014-0201-2 PMC4267430

[34] Bauman DE, Currie WB. Partitioning of nutrients during pregnancy and lactation: A review of mechanisms involving homeostasis and homeorhesis. J Dairy Sci. 1980;63: 1514–1529. 7000867 10.3168/jds.s0022-0302(80)83111-0

[35] Smith NE, Baldwin RL. Effects of breed, pregnancy, and lactation on weight of organs and tissues in dairy cattle. J Dairy Sci. 1974;57: 1055.

[36] Baldwin RL, McLeod KR, Capuco AV. Visceral tissue growth and proliferation during the bovine lactation cycle. J Dairy Sci. 2004;87: 2977–2986. doi: 10.3168/jds.S0022-0302(04)73429-3 15375059 10.3168/jds.S0022-0302(04)73429-3

[37] McLeod KR, Baldwin RL. Effects of diet forage:concentrate ration and metabolizable energy intake on visceral organ growth and in vitro oxidative capacity of gut tissues in sheep. J Anim Sci. 2000;78: 760–770. 10764085 10.2527/2000.783760x

[38] Hauso Ø, Gustafsson BI, Loennechen JP, Stunes AK, Nordrum I, Waldum HL. Long-term serotonin effects in the rat are prevented by terguride. Reg Pept. 2007;143: 39–46. 10.1016/j.regpep.2007.02.009 17391782

[39] Albrecht JH, Hansen LK. Cyclin D1 promotes mitogen-independent cell cycle progression in hepatocytes. Cell Growth Differ. 1999;10: 397–404. 10392901

[40] Paunesku T, Mittal S, Protić M, Oryhon J, Korolev SV, Joachimiak A, Woloschak GE. Proliferating cell nuclear antigen (PCNA): ringmaster of the genome. Int J Radiat Biol. 2001;77: 1007–1021. doi: 10.1080/09553000110069335 11682006 10.1080/09553000110069335

[41] Collier RJ, Annen-Dawson EL, Pezeshki A. Effects of continuous lactation and short dry periods on mammary function and animal health. Animal 2012;6: 403–414. doi: 10.1017/S1751731111002461 22436219 10.1017/S1751731111002461

[42] Laubenthal L, Hoelker M, Frahm J, Dänicke S, Gerlach K, Südekum KH, et al Mitochondrial DNA copy number and biogenesis in different tissues of early- and late-lactating dairy cows. J Dairy Sci. 2016;99: 1571–1583. doi: 10.3168/jds.2015-9847 26686730 10.3168/jds.2015-9847

[43] Tharwat M, Takamizawa A, Hosaka YZ, Endoh D, Oikawa S. Hepatocyte apoptosis in dairy cattle during the transition period. Can J Vet Res. 2012a;76: 241–247. 23543948 PMC3460600

[44] Tharwat M, Endoh D, Oikawa S. Hepatocyte apoptosis in cows with fatty infiltration of the liver. Res Vet Sci. 2012b;93: 1281–1286. 22513126 10.1016/j.rvsc.2012.03.011

[45] White HM, Charvalho ER, Koser SL, Schmelz-Roberts NS, Pezzanite LM, Slabaugh AC, et al Short communication: Regulation of hepatic gluconeogenic enzymes by dietary glycerol in transition dairy cows. J Dairy Sci. 2016;99: 812–817. doi: 10.3168/jds.2015-9953 26547649 10.3168/jds.2015-9953

[46] Al-Trad B, Wittek T, Penner GB, Reisberg K, Gäbel G, Fürll M, Aschenbach JR. Expression and activity of key hepatic gluconeogenesis enzymes in response to increasing intravenous infusions of glucose in dairy cows. J Anim Sci. 2010;88: 2998–3008. doi: 10.2527/jas.2009-2463 20495114 10.2527/jas.2009-2463

[47] Donkin SS, Armentano LE. Insulin and glucagon regulation of gluconeogenesis in preruminating and ruminating bovine. J Anim Sci 1995;73: 546–551. 7601789 10.2527/1995.732546x

[48] White HM. The role of TCA anaplerosis in ketosis and fatty liver in periparturient dairy cows. Animals (Basel) 2015;18: 793–802. 10.3390/ani5030384 PMC4598706 26479386

[49] Walsh RB, Walton JS, Kelton DF, LeBlanc SJ, Leslie KE, Duffield TF. The effect of subclinical ketosis in early lactation on reproductive performance of postpartum dairy cows. J Dairy Sci. 2007;90: 2788–2796. doi: 10.3168/jds.2006-560 17517719 10.3168/jds.2006-560

[50] Li Y, Ding HY, Wang XC, Feng SB, Li XB, Wang Z, et al An association between the level of oxidative stress and the concentrations of NEFA and BHBA in the plasma of ketotic dairy cows. J Anim Physiol Anim Nutr (Berl). 2016;100: 844–851. 27079290 10.1111/jpn.12454

[51] Martinez N, Risco CA, Lima FS, Bisinotto RS, Greco LF, Ribeiro ES, et al Evaluation of peripartal calcium status, energetic profile, and neutrophil function in dairy cows at low or high risk of developing uterine disease. J Dairy Sci. 2012;95: 7158–7172. doi: 10.3168/jds.2012-5812 23021755 10.3168/jds.2012-5812

[52] Wylie ARG, Woods S, Carson AF, McCoy M. Periprandial changes in metabolite and metabolic hormone concentrations in high-genetic-merit dairy heifers and their relationship to energy balance in early lactation. J Dairy Sci. 2008;91: 577–586. doi: 10.3168/jds.2007-0388 18218744 10.3168/jds.2007-0388

[53] French PD. Dry matter intake and blood parameters of nonlactating Holstein and Jersey cows in late lactation. J Dairy Sci. 2006;89: 1057–1061. doi: 10.3168/jds.S0022-0302(06)72173-7 16507702 10.3168/jds.S0022-0302(06)72173-7

[54] Moore SA, Laporta J, Crenshaw TD, Hernandez LL. Patterns of circulating serotonin and related metabolites in multiparous dairy cows in the peripartum period. J Dairy Sci. 2015;98: 3754–3765. doi: 10.3168/jds.2014-8841 25828664 10.3168/jds.2014-8841

[55] Olson KM, Cassell BG, Hanigan MD, Pearson RE. Interaction of energy balance, feed efficiency, early lactation health events, and fertility in first lactation Holstein, Jersey, and reciprocal F1 crossbred cows. J Dairy Sci. 2011;94: 507–511. doi: 10.3168/jds.2010-3433 21183063 10.3168/jds.2010-3433

