# Supplementary figures and images for: Elevating serotonin pre-partum alters the Holstein dairy cow hepatic adaptation to lactation
Source: PLoS One. 2017 Sep 18;12(9):e0184939. doi: 10.1371/journal.pone.0184939 (PMC5602632; doi:10.1371/journal.pone.0184939)

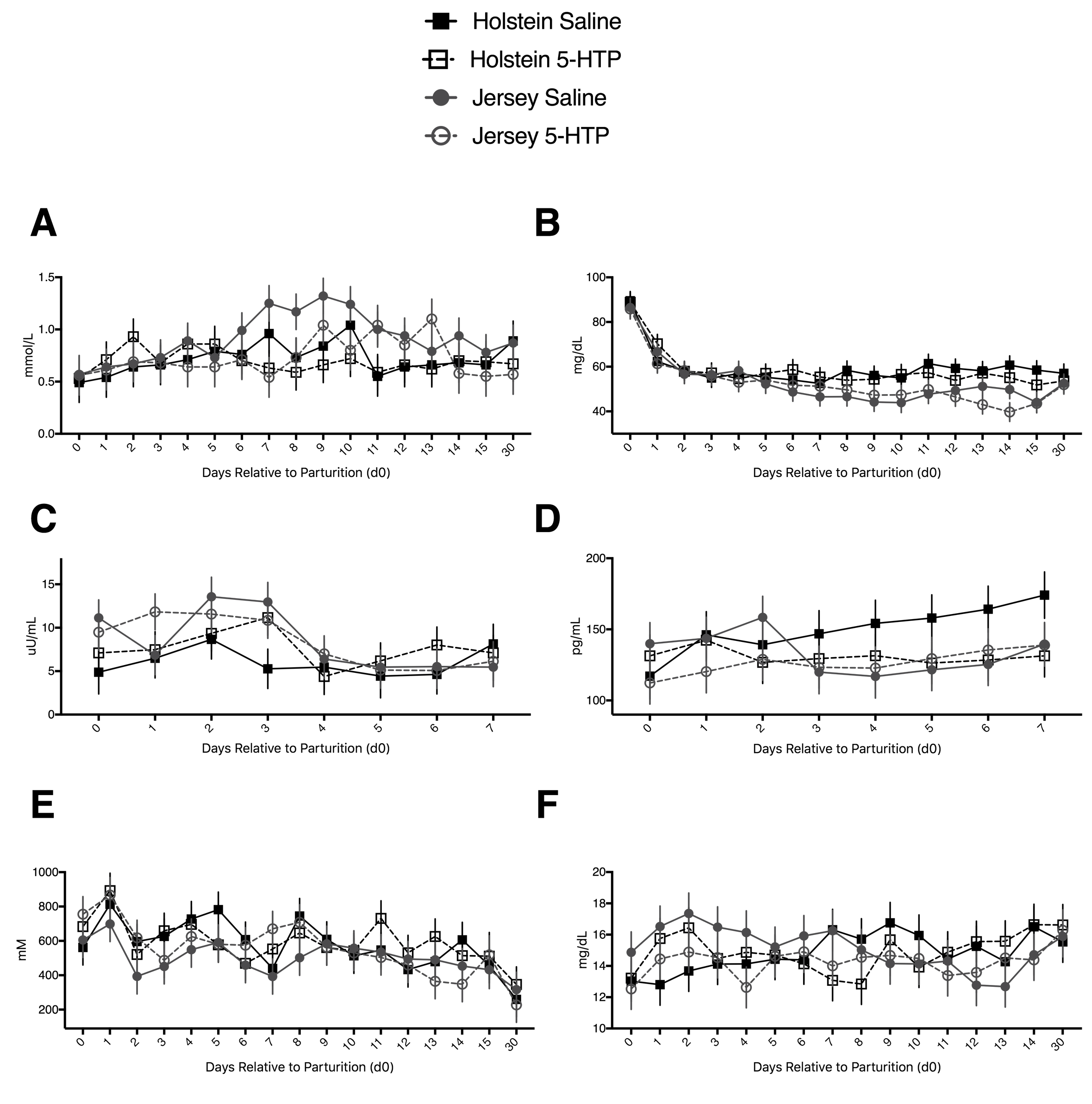

Supplement: S1 Fig — Final treatment groups were saline-infused Holsteins (n = 6), 5-HTP infused Holsteins (n = 6), saline-infused Jerseys (n = 6), and 5-HTP infused Jerseys (n = 6). On average, Holstein cows were infused for 5.67 ± 0.78 days and Jersey cows were infused for 8.67 ± 1.53 days. Profiles of (A) beta-hydroxybutyrate (BHBA) (B) glucose (C) insulin (D) glucagon (E) non-esterified fatty acid (NEFA) and (F) plasma urea nitrogen (PUN) levels in either plasma or serum for either 7 or 14 days and on d30 post-partum. All values are reported as LS means ± SEM. (TIFF) [file pone.0184939.s001.tiff]
