# Supplementary material for: Elevating serotonin pre-partum alters the Holstein dairy cow hepatic adaptation to lactation
Source: PLoS One. 2017 Sep 18;12(9):e0184939. doi: 10.1371/journal.pone.0184939 (PMC5602632; doi:10.1371/journal.pone.0184939)
Supplement: S1 Table — (DOCX) [file pone.0184939.s002.docx]

| **Metabolite** | **Trt** | **DRP** | **Brd** | **Trt*DRP** | **Trt*Brd** | **Brd*DRP** | **Trt*Brd*DRP** |
| --- | --- | --- | --- | --- | --- | --- | --- |
| **BHBA** | *P*=0.31 | *P*=0.11 | *P*=0.27 | *P*=0.02 | *P*=0.43 | *P*=0.12 | *P*=0.64 |
| **Glucose** | *P*=0.74 | *P*<0.0001 | *P*=0.003 | *P*=0.89 | *P*=0.98 | *P*=0.84 | *P*=0.91 |
| **Insulin** | *P*=0.48 | *P*<0.0001 | *P*=0.15 | *P*=0.92 | *P*=0.58 | *P*=0.20 | *P*=0.82 |
| **Glucagon** | *P*=0.31 | *P*=0.02 | *P*=0.39 | *P*=0.80 | *P*=0.62 | *P*=0.05 | *P*=0.01 |
| **NEFA** | *P*=0.50 | *P*<0.0001 | *P*=0.24 | *P*=0.80 | *P*=0.71 | *P*=0.91 | *P*=0.71 |
| **PUN** | *P*=0.60 | *P*=0.06 | *P*=0.83 | *P*=0.53 | *P*=0.54 | *P*=0.60 | *P*=0.66 |

Main effects and their interactions are listed as headers of each column. Trt – treatment, DRP – days relative to parturition, Brd – breed. An asterisk between main effects indicates the test of their interaction (i.e.: Trt*DRP – the interaction of treatment with days relative to parturition). The metabolites tested are listed in each row. BHBA – beta-hydroxybutyrate, NEFA – non-esterified fatty acids, PUN – plasma urea nitrogen.
