# Supplementary material for: Elevating serotonin pre-partum alters the Holstein dairy cow hepatic adaptation to lactation
Source: PLoS One. 2017 Sep 18;12(9):e0184939. doi: 10.1371/journal.pone.0184939 (PMC5602632; doi:10.1371/journal.pone.0184939)
Supplement: S2 Table — (DOCX) [file pone.0184939.s003.docx]

| **Metabolite** | **Brd** | **Trt** | **DRP** | **Trt*DRP** | **Brd** | **Trt Effect** | **DRP** | **Trt*DRP** |
| --- | --- | --- | --- | --- | --- | --- | --- | --- |
| **BHBA** | **Holstein** | *P*=0.84 | *P*=0.008 | *P*=0.40 | **Jersey** | *P*=0.29 | *P*=0.39 | *P*=0.10 |
| **Glucose** |  | *P*=0.78 | *P*<0.0001 | *P*=0.90 |  | *P*=0.84 | *P*<0.0001 | *P*=0.90 |
| **Insulin** |  | *P*=0.38 | *P*=0.31 | *P*=0.60 |  | *P*=0.97 | *P*=0.0004 | *P*=0.78 |
| **Glucagon** |  | *P*=0.44 | *P*=0.002 | *P*=0.07 |  | *P*=0.53 | *P*=0.16 | *P*=0.28 |
| **NEFA** |  | *P*=0.79 | *P*<0.0001 | *P*=0.81 |  | *P*=0.53 | *P*=0.0006 | *P*=0.73 |
| **PUN** |  | *P*=0.96 | *P*=0.11 | *P*=0.41 |  | *P*=0.37 | *P*=0.35 | *P*=0.75 |

Each breed (Brd) is separated within the table. Main effects and their interactions are listed as headers of each column. Trt – treatment, DRP – days relative to parturition. An asterisk between main effects indicates the test of their interaction (i.e.: Trt*DRP – the interaction of treatment with days relative to parturition). The metabolites tested are listed in each row. BHBA – beta-hydroxybutyrate, NEFA – non-esterified fatty acids, PUN – plasma urea nitrogen.
