# Supplementary material for: Elevating serotonin pre-partum alters the Holstein dairy cow hepatic adaptation to lactation
Source: PLoS One. 2017 Sep 18;12(9):e0184939. doi: 10.1371/journal.pone.0184939 (PMC5602632; doi:10.1371/journal.pone.0184939)
Supplement: S3 Table — (DOCX) [file pone.0184939.s004.docx]

| **Metabolite** | **Trt** | **Brd** | **DRP** | **Brd*DRP** | **Trt** | **Brd** | **DRP** | **Brd*DRP** |
| --- | --- | --- | --- | --- | --- | --- | --- | --- |
| **BHBA** | **Saline** | *P*=0.30 | *P*=0.10 | *P*=0.97 | **5-HTP** | *P*=0.72 | *P*=0.03 | *P*=0.003 |
| **Glucose** |  | *P*=0.03 | *P*<0.0001 | *P*=0.86 |  | *P*=0.04 | *P*<0.0001 | *P*=0.90 |
| **Insulin** |  | *P*=0.10 | *P*=0.13 | *P*=0.31 |  | *P*=0.64 | *P*=0.004 | *P*=0.42 |
| **Glucagon** |  | *P*=0.48 | *P*=0.17 | *P*=0.02 |  | *P*=0.60 | *P*=0.50 | *P*=0.20 |
| **NEFA** |  | *P*=0.22 | *P*=0.0003 | *P*=0.83 |  | *P*=0.61 | *P*<0.0001 | *P*=0.81 |
| **PUN** |  | *P*=0.70 | *P*=0.47 | *P*=0.48 |  | *P*=0.63 | *P*=0.10 | *P*=0.78 |

Each treatment (Trt) is separated within the table. Main effects and their interactions are listed as headers of each column. Brd – breed, DRP – days relative to parturition. An asterisk between main effects indicates the test of their interaction (i.e.: Brd*DRP – the interaction of breed with days relative to parturition). The metabolites tested are listed in each row. BHBA – beta-hydroxybutyrate, NEFA – non-esterified fatty acids, PUN – plasma urea nitrogen.
